# Supplementary material for: Pre-meiotic H1.1 degradation is essential for Arabidopsis gametogenesis
Source: EMBO J. 2026 Jan 3;45(3):789–819. doi: 10.1038/s44318-025-00671-2 (PMC12864861; doi:10.1038/s44318-025-00671-2)
Supplement: Supplementary file 14 — Expanded View Figures [file 44318_2025_671_MOESM14_ESM.pdf]

# Expanded View Figures

**Figure EV1. H1.1 degradation is controlled by CUL4 and the K89 residue, without altering its stability.**

(**A**) Relative Expression of CUL1 (AT4G02570), CUL2 (AT1G02980), CUL3A (AT1G26830), CUL3B (AT1G69670), CUL4 (AT5G46210) in different tissue present in Affymetrix ATH1 GeneCHIP experiments from publicly available datasets (Wuest et al, 2010; Borges et al, 2008; Honys and Twell, 2004; Pina et al, 2005; Schmidt et al, 2011a). The dendrogram in the expression heatmap was generated using hierarchical clustering with default options in the heatmap.2 function from the gplots package in R (R Core Team, 2023; Warnes et al, 2024). Pairwise distances were calculated using the Euclidean distance metric, and hierarchical clustering was performed using the complete linkage method. These settings were used to cluster both rows and columns. The Shiny App used to generate the heatmap is available at https://github.com/AleGirFon/Arabidopsis_heatmap. The table with normalized expression values can be found in Source Data EV1A. Abbreviation: MMC (megaspore mother cell), EmbryoHea (embryo heart stage), EmbryoPreGlo (embryo pre-globular stage). (**B**) Schematic representation of *CULLIN4* (At5g46210) genomic region with indication of the position and sequence of the artificial micro-RNA used in *amiR[CUL4]* lines. (**C**) Western blot (left panel) detection using an anti-CUL4 antibody and Ponceau staining below in *amiRNA [CUL4]* Line #8, treated with Dex and mock, each in two independent replicate extractions. Total proteins were extracted from seedlings at 12 DPI, loaded and immunoblotted as described in the Methods. The expected molecular weight of CULLIN 4 is indicated (~91 KDa). Right: quantification of the relative CUL4 band intensity (see Methods) in three independent lines (4, #7 and #8), each in two replicate protein extraction. Blots used for quantifications in Source Data EV1C. *P* value: Mann–Whitney U test. Boxplots: Center lines indicate medians; boxes span the interquartile range (25th–75th percentiles); whiskers extend 1.5× the interquartile range beyond the box limits, as computed in R. (**D**) *CUL4* knockdown as in the Dex inducible *amiRNA[CUL4]* line recapitulates a known f*usca* phenotype (Chen et al, 2006). Seedlings are 11DAG-old and were grown on ½ MS supplemented with 10 µM DEX (upper panel) or a MOCK solution (bottom panel). Whit arrows indicate brownish pigmentation in the cotyledons, representing the f*usca* phenotype, Scale bar = 5 mm. (**E**) Screen shot from the Plant PTM Viewer webserver. The Plant PTM Viewer shows six putative ubiquitination sites on Arabidopsis H1.1 (Willems et al, 2019). (**F**) Representative images of H1.1-RFP, H1.1$^{6xGC}$-RFP in a wild-type background and co-expressed with H1.1-GFP under its native promoter (She et al, 2013) showing persistence of H1.1$^{6xGC}$-RFP and depletion of H1.1-GFP in the SMC (dashed lines) of ovule primordia 5 dpi; Dotted border outlines the SMC. Pie charts show replicate measurements of persistence vs depletion categories in independent lines. n: number of scored ovule primordia. *P* values, Fisher exact test. (**G**) Fluorescent Recovery After Photobleaching experiments measuring the mobility of the different H1.1 variants as indicated in seedling roots and Boxplot showing the recovery rate at 30 s, 60s- and 4-min. *n*: number of analyzed nuclei; error bar: standard error to mean. *P* values, Mann–Whitney U test. Boxplots: Center lines indicate medians; boxes span the interquartile range (25th–75th percentiles); whiskers extend 1.5× the interquartile range beyond the box limits, as computed in R. (**H**) Alignment of selected Arabidopsis and mouse H1 variants (AtH1.1: AT1G06760 H1.1; AtH1.2: AT2G30620 H1.2, Mouse H1.0: P10922, Mouse H1.1: P43275, Mouse H1.2: P15864) cropped around the globular domain (yellow) showing the position of the conserved Lysin residue (K89 in AtH1.1) and the three α helices as indicated. Right: representation of AtH1.1 3D folding of the globular domain, indicating the position of K89. (**I**) Replicate measurements of H1.1$^{K89R}$ persistence vs depletion in the SMC of independent lines induced as described in the main text and compared to a control line. Dotted border outlines the SMC. *P* value: Fisher exact test. (**J**) 3D projection showing H1.1$^{6GC}$-RFP and H1.1$^{R57K}$-RFP persistence in both euchromatin and heterochromatin. Top panel: representation of the image processing used to isolate nuclei in silico for projections (left, whole primordium counterstained with Renaissance; middle, primordium after segmentation and masking of the SMC nucleus (magenta) and several nucellus nuclei (cyan); right: nuclei projection only). Middle and bottom panels: three representative images for the H1.1 variants as indicated. Scale bar: 5 µm. (**K**) Sequence alignment of the forward (F) and reverse (R) sequencing fragments from the transgenes of 12 independent transgenic lines (DLF15-26) covering the H1.1 transgene region revealed A to G mutations, enabling eleven K to R substitutions, including at residue K89. Images below show low H1.1-11G-RFP signals in ovule primordia in comparison to H1.1-6xGC-RFP. Dotted border outlines the SMC. See also Source data Fig. EV1 and Table EV1. Source data are available online for this figure.

 © The Author(s)

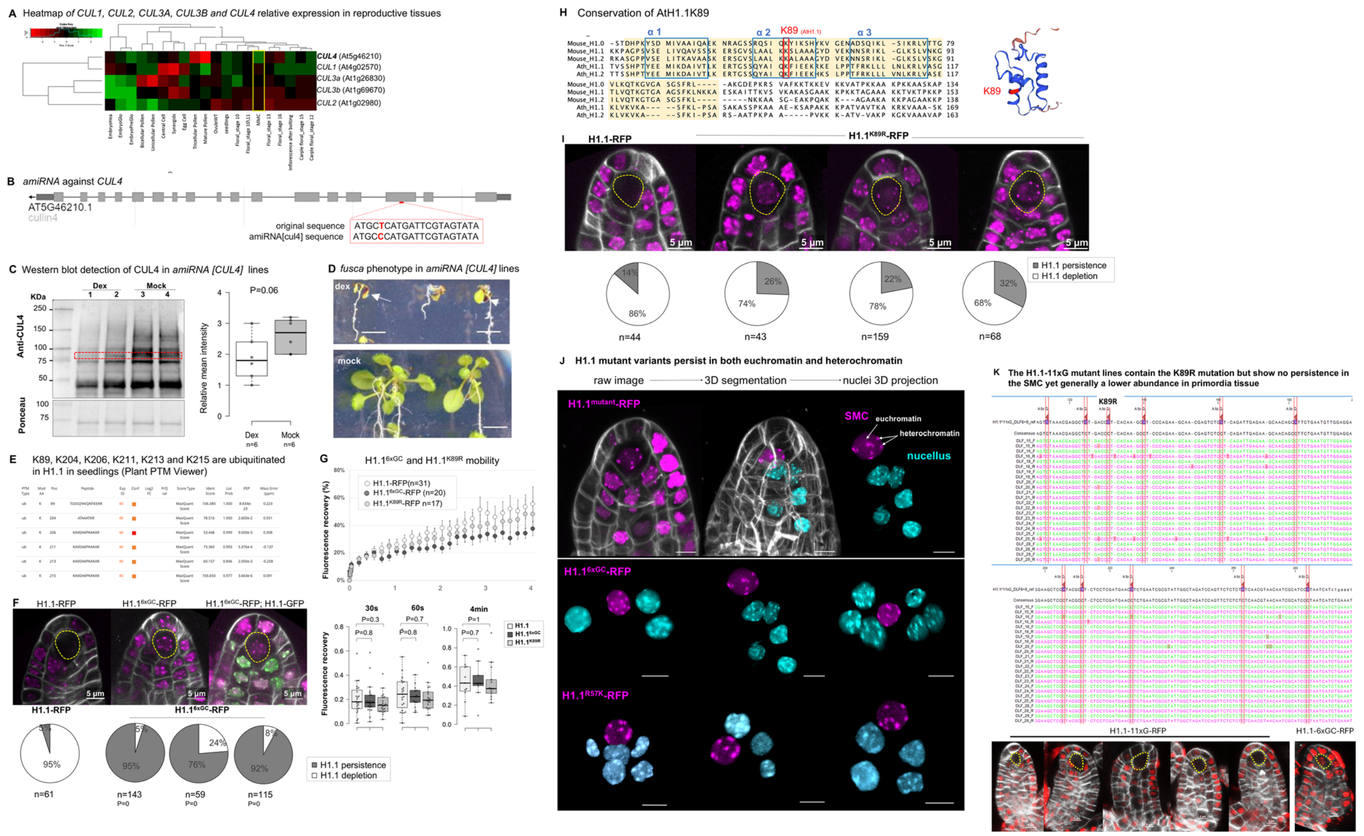
A Heatmap of CUL1, CUL2, CUL3A, CUL3B and CUL4 relative expression in reproductive tissues
CUL4 (At5g46210)
CUL1 (At4g02570)
CUL3a (At1g26830)
CUL3b (At1g69670)
CUL2 (At1g02980)
B amiRNA against CUL4
AT5G46210.1
cullin4
original sequence ATGCTCATGATTCGTAGTATA
amiRNA[cul4] sequence ATGCCCATGATTCGTAGTATA
C Western blot detection of CUL4 in amiRNA [CUL4] lines
Dex
Mock
Anti-CUL4
Ponceau
P=0.06
Relative mean intensity
D fusca phenotype in amiRNA [CUL4] lines
dex
mock
E K89, K204, K206, K211, K213 and K215 are ubiquitinated in H1.1 in seedlings (Plant PTM Viewer)
F H1.1-RFP
H1.1^6xGC-RFP
H1.1^6xGC-RFP; H1.1-GFP
5 µm
95%
76%
92%
24%
n=61
n=143
n=59
n=115
H1.1 persistence
H1.1 depletion
G H1.1^6xGC and H1.1^K89R mobility
H1.1-RFP(n=31)
H1.1^6xGC-RFP (n=20)
H1.1^K89R-RFP n=17)
Fluorescence recovery (%)
30s
60s
4min
H Conservation of AtH1.1K89
α 1
α 2
α 3
K89
I H1.1-RFP
H1.1^K89R-RFP
14%
86%
26%
74%
22%
78%
32%
68%
n=44
n=43
n=159
n=68
H1.1 persistence
H1.1 depletion
J H1.1 mutant variants persist in both euchromatin and heterochromatin
raw image
3D segmentation
nuclei 3D projection
H1.1^mutant-RFP
SMC
euchromatin
heterochromatin
nucellus
H1.1^6xGC-RFP
H1.1^R57K-RFP
K The H1.1-11xG mutant lines contain the K89R mutation but show no persistence in the SMC yet generally a lower abundance in primordia tissue
K89R
H1.1-11xG-RFP
H1.1-6xGC-RFP

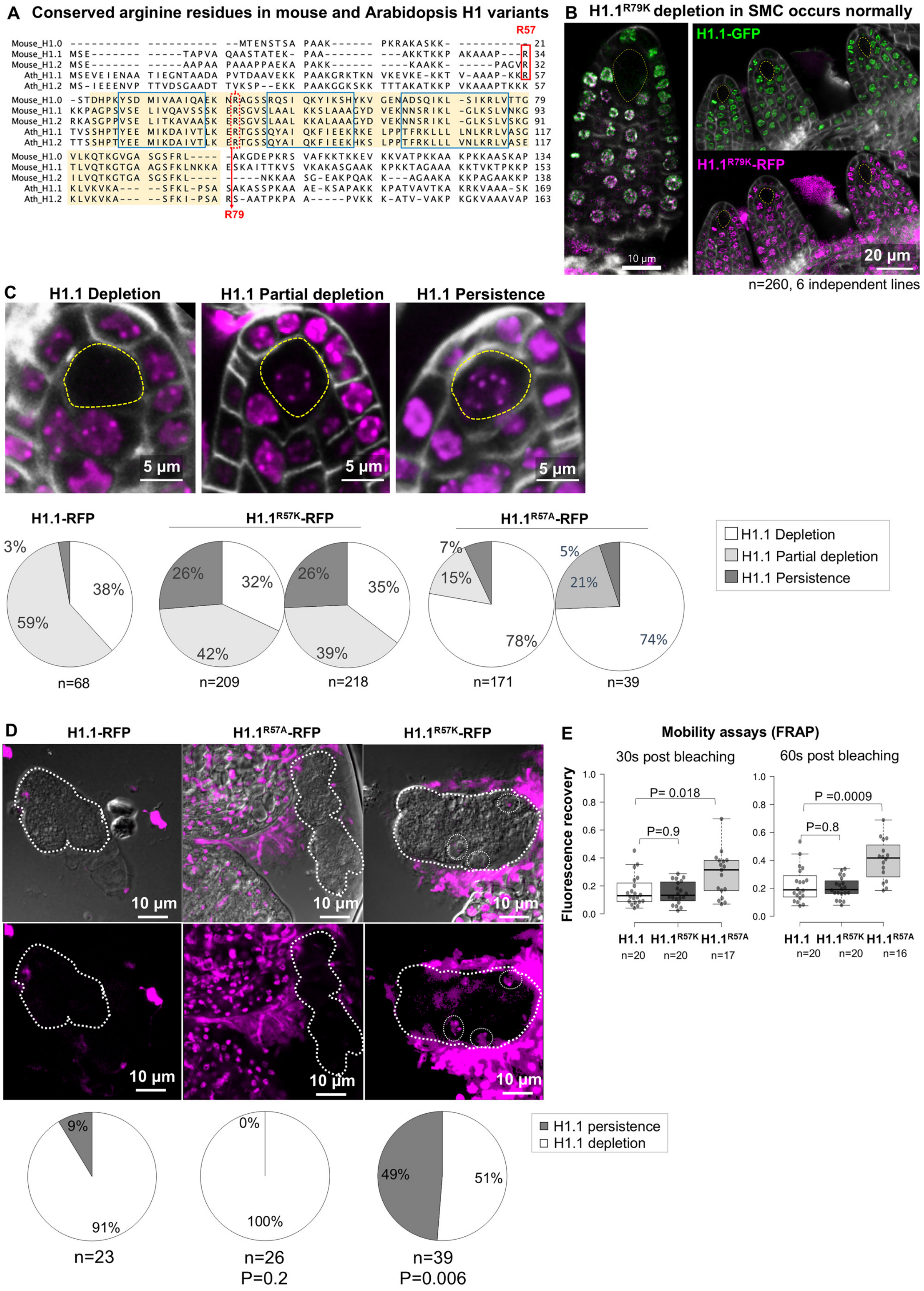
A Conserved arginine residues in mouse and Arabidopsis H1 variants
R57
R79
B H1.1R79K depletion in SMC occurs normally
H1.1-GFP
H1.1R79K-RFP
10 µm
20 µm
n=260, 6 independent lines
C
H1.1 Depletion
H1.1 Partial depletion
H1.1 Persistence
5 µm
H1.1-RFP
H1.1R57K-RFP
H1.1R57A-RFP
3% 38% 59%
n=68
26% 32% 42%
n=209
26% 35% 39%
n=218
7% 15% 78%
n=171
5% 21% 74%
n=39
H1.1 Depletion
H1.1 Partial depletion
H1.1 Persistence
D
H1.1-RFP
H1.1R57A-RFP
H1.1R57K-RFP
10 µm
9% 91%
n=23
0% 100%
n=26
P=0.2
49% 51%
n=39
P=0.006
H1.1 persistence
H1.1 depletion
E Mobility assays (FRAP)
30s post bleaching
60s post bleaching
Fluorescence recovery
P= 0.018
P=0.9
P =0.0009
P=0.8
H1.1 H1.1R57K H1.1R57A
n=20 n=20 n=17
n=20 n=20 n=16

 © The Author(s)

◀ **Figure EV2. H1.1 depletion in the SMC is controlled by the R57 residue (related to Fig. 2).**

(**A**) Alignment of selected Arabidopsis and Mouse variants as those in Fig. EV1 (cropped around the globular domain, yellow, with parts of the N- and C-tails) showing the conservation of arginine residues (red boxes). The R57 residue from AtH1.1 studied in this work is positioned in the N-tail just before the globular domain. It is conserved in the Mouse H1.1 and H1.2 variants but not in the Arabidopsis H1.2 variant. The arginine shown by Christophorou and colleagues to be citrullinated is R54 in the Mouse H1.2 variant corresponds to R79 in AtH1.1 and AtH1.2. (**B**) H1.1$^{R79K}$-RFP (magenta) is normally depleted in SMCs (dotted outline), following the native H1.1-GFP variant (green). Ovule primordia 5 dpi were counterstained with Renaissance (gray). (**C**) Distribution patterns of H1.1 mutant variants showing either full depletion, partial depletion and persistence in the SMC (dotted outline) of ovule primordia stage 1-II/2-I at 5 dpi (partial confocal projections are shown in the images) as used for scoring shown in the pie charts for the H1.1-RFP control line and two independent lines expressing the mutant variants as indicated. *n*, number of primordia scored. The images illustrating H1.1 Depletion and H1.1 Persistence are reused from Fig. 2B for facilitating comparisons. See *P* values from a chi2 contingency test comparing the distribution of the three categories Table EV1. (**D**) In premeiotic sporangia from flowers at 3 dpi, H1.1-RFP and H1.1$^{R57A}$-RFP are depleted in male SMC (pollen mother cells, dotted outline), but H1.1$^{R57K}$-RFP shows residual signal (magenta). Confocal images show overlays of RFP signal (magenta) with transmission light images with differential interference contrast (DIC); pie charts quantify the observations. *n*, number of sporangia. Dotted border outlines Pollen Mother Cells (PMC). (**E**) Fluorescence recovery rate from FRAP experiments shown Fig. 2, at 30 s and 60 s post bleaching. *n*: number of analyzed nuclei. Boxplots: Center lines indicate medians; boxes span the interquartile range (25th–75th percentiles); whiskers extend 1.5× the interquartile range beyond the box limits, as computed in R. *P* value, Mann–Whitney U test. See also Source data Fig. EV2 and Table EV1. Source data are available online for this figure.

© The Author(s) 

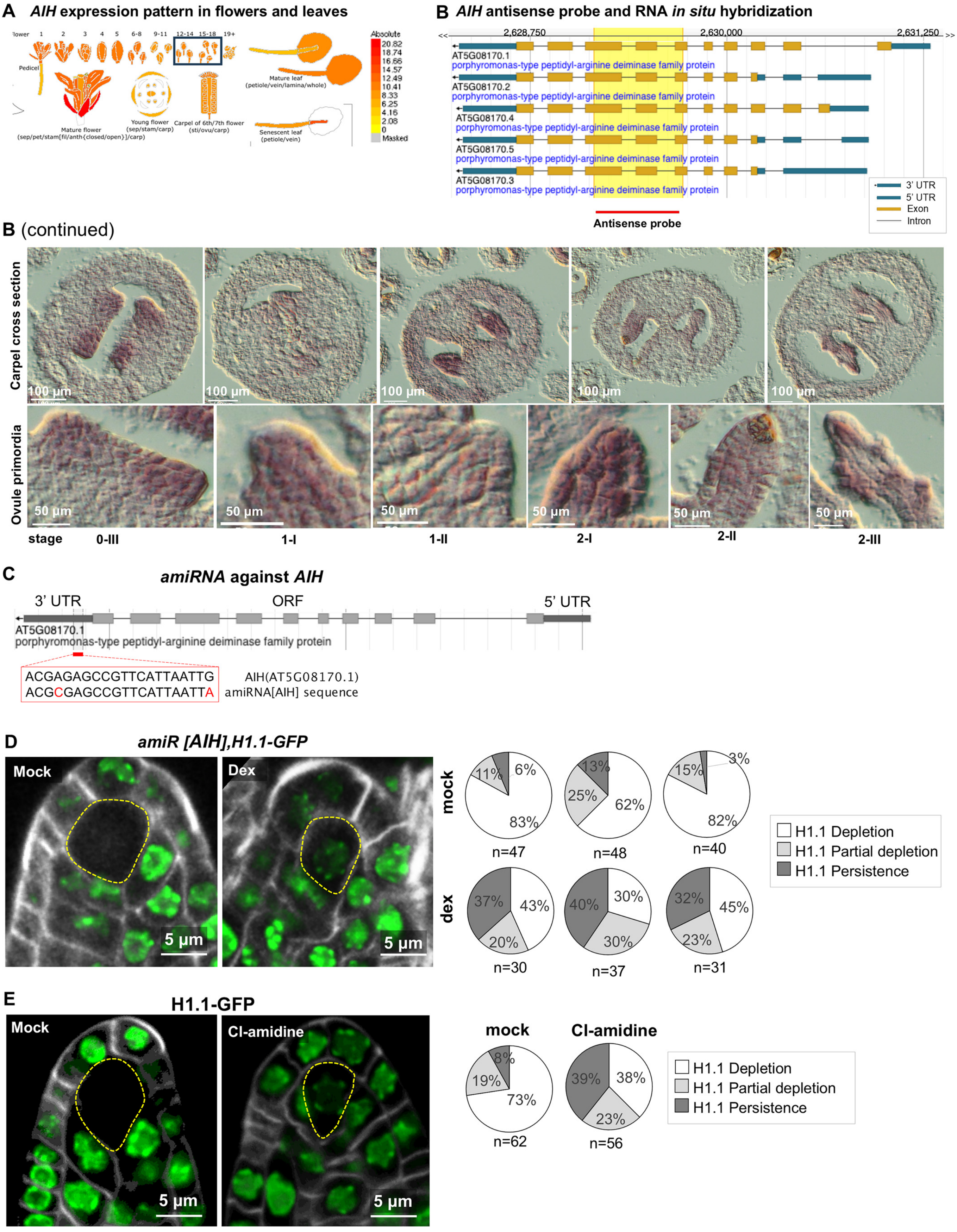
A AIH expression pattern in flowers and leaves
B AIH antisense probe and RNA in situ hybridization
AT5G08170.1
porphyromonas-type peptidyl-arginine deiminase family protein
AT5G08170.2
AT5G08170.4
AT5G08170.5
AT5G08170.3
3' UTR
5' UTR
Exon
Intron
Antisense probe
B (continued)
Carpel cross section
Ovule primordia
100 μm
50 μm
stage 0-III 1-I 1-II 2-I 2-II 2-III
C amiRNA against AIH
3' UTR ORF 5' UTR
ACGAGAGCCGTTCATTAATTG AIH(AT5G08170.1)
ACGCGAGCCGTTCATTAATTA amiRNA[AIH] sequence
D amiR [AIH],H1.1-GFP
Mock
Dex
5 μm
mock
dex
6% 11% 83% n=47
13% 25% 62% n=48
3% 15% 82% n=40
37% 43% 20% n=30
40% 30% 30% n=37
32% 45% 23% n=31
H1.1 Depletion
H1.1 Partial depletion
H1.1 Persistence
E H1.1-GFP
Mock
Cl-amidine
5 μm
mock
Cl-amidine
8% 19% 73% n=62
39% 38% 23% n=56
H1.1 Depletion
H1.1 Partial depletion
H1.1 Persistence

 © The Author(s)

**Figure EV3. The AIH citrullinase mediates H1.1 depletion in SMC (related to Fig. 3).**

(**A**) Selected view of *AIH* expression generated by the ePlant Browser (bar.utoronto.ca/eplant/) showing middle-to-strong expression in young flower buds (box). (**B**) Schematic representation of the five splice variants of *AIH* (TAIR resource, arabidopsis.org) and the position of the probe used for RNA in situ hybridization shown below. Top panel: cross section through carpels, Bottom panel: Magnified views of the top panel, illustrating ovule primordia from the corresponding carpels at the indicated developmental stages (0-III, 2-III) following the nomenclature (Hernandez-Lagana et al, 2021). The third image in the top panel and the stage 2-I image in the bottom panel are reused from Fig. 3B to facilitate comparisons. (**C**) Position and sequence of the amiRNA used for downregulating *AIH*. (**D**, **E**) Representative images and scoring showing the effect of *AIH* downregulation using an amiRNA (**D**) or inhibition using Cl-amidine (**E**). Top panels: images of mock and Dex (**D**), or mock and Cl-amidine (**E**) treated ovule primordia at 5 dpi expressing H1.1-GFP under its native promoter (She et al, 2013) and the inducible *amiR[AIH]* as described in the main text. Dotted contours outline the SMC. Pie charts: replicate scoring of depletion/partial depletion/persistence pattern of H1.1-GFP in the SMC, in three independent lines. *n*, number of ovule primordia scored, *P* values testing the distribution of all three categories: see Table EV1. Confocal images in (**D**) and (**E**) are reused from Figs. 3C and 3D, respectively, for facilitating comparisons. See also Source data Fig. EV3 and Table EV1. Source data are available online for this figure.

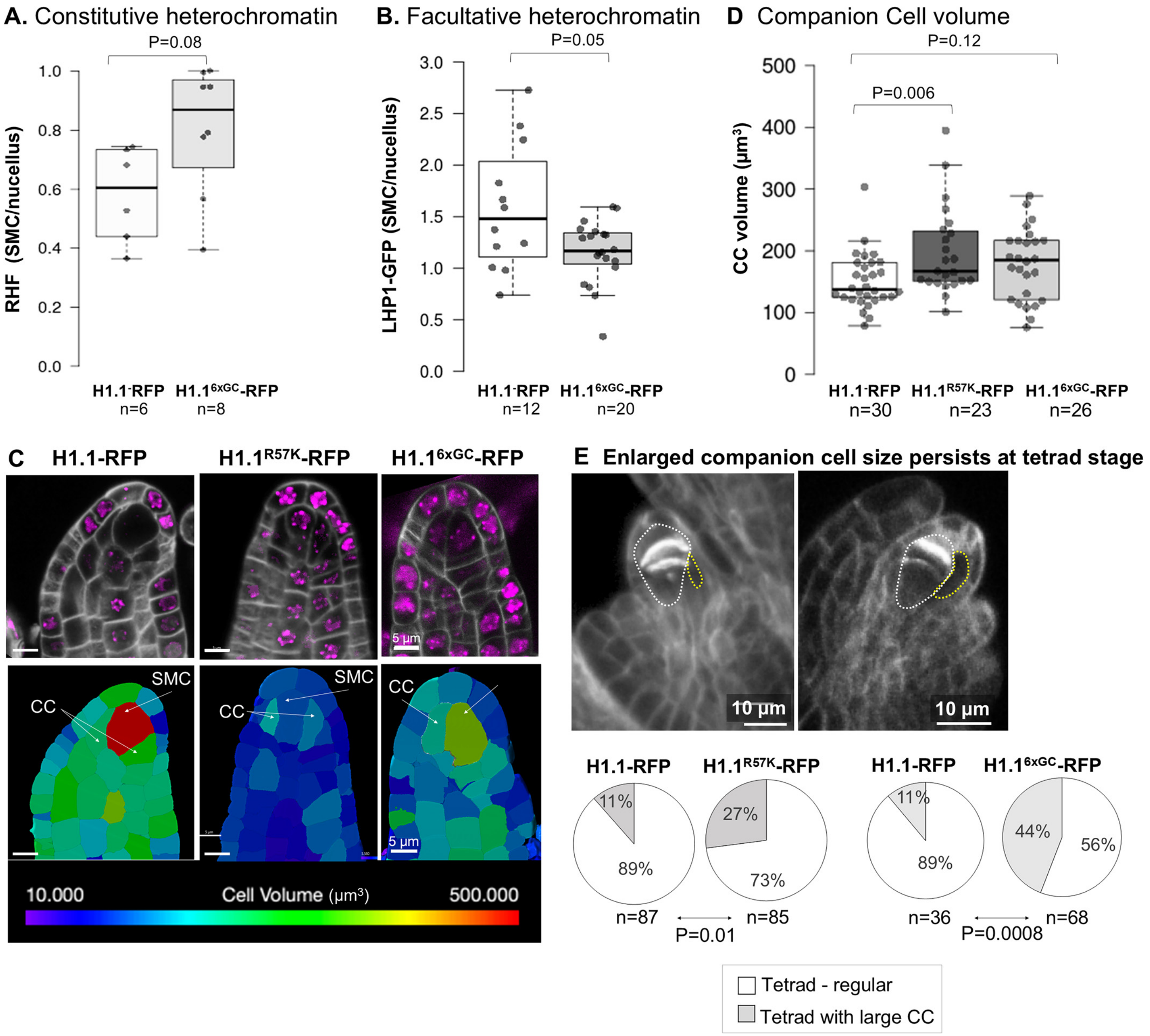


**Figure EV4. Effect of H1.1 persistence in the SMC on chromatin, SMC maturation and meiosis (related to Fig. 4).**

(**A**, **B**) Relative Heterochromatin Fraction (RHF, **A**) and LHP1-GFP levels (**B**) in the SMC relative to surrounding nucellar cells, in the H1.1-RFP control line and H1.1$^{6xGC}$-RFP mutant line. (**C**) Representative images and corresponding cell-based segmentation (below) of ovule primordia at 5 dpi expressing the control of mutant H1.1 variants as indicated, to measure the SMC and CC volumes as plotted in Fig. 4 and Panel D here. The segmented image for H1.1-RFP is reused from Fig. 4D to facilitate comparisons. (**D**) Volume of the companion cells (CC) of ovule primordia at 5 dpi expressing the control or mutant H1.1 variants as indicated. (**E**) Representative images of ovules at the tetrad stage showing a tetrad (white dotted line) and a neighboring, narrow or enlarged CC (left and right, respectively, yellow dotted line). Pie charts showing scoring of the respective classes in ovule primordia at 6 dpi, induced for the expression of the control or mutant H1.1 variants as indicated. *n*, number of ovule primordia scored. *P* values, Mann–Whitney U test (**A**, **B**, **D**) or Fisher exact test (**C**). Boxplots: Center lines indicate medians; boxes span the interquartile range (25th–75th percentiles); whiskers extend 1.5× the interquartile range beyond the box limits, as computed in R. See also Source data Fig. EV4 and Table EV1. Source data are available online for this figure.

 © The Author(s)

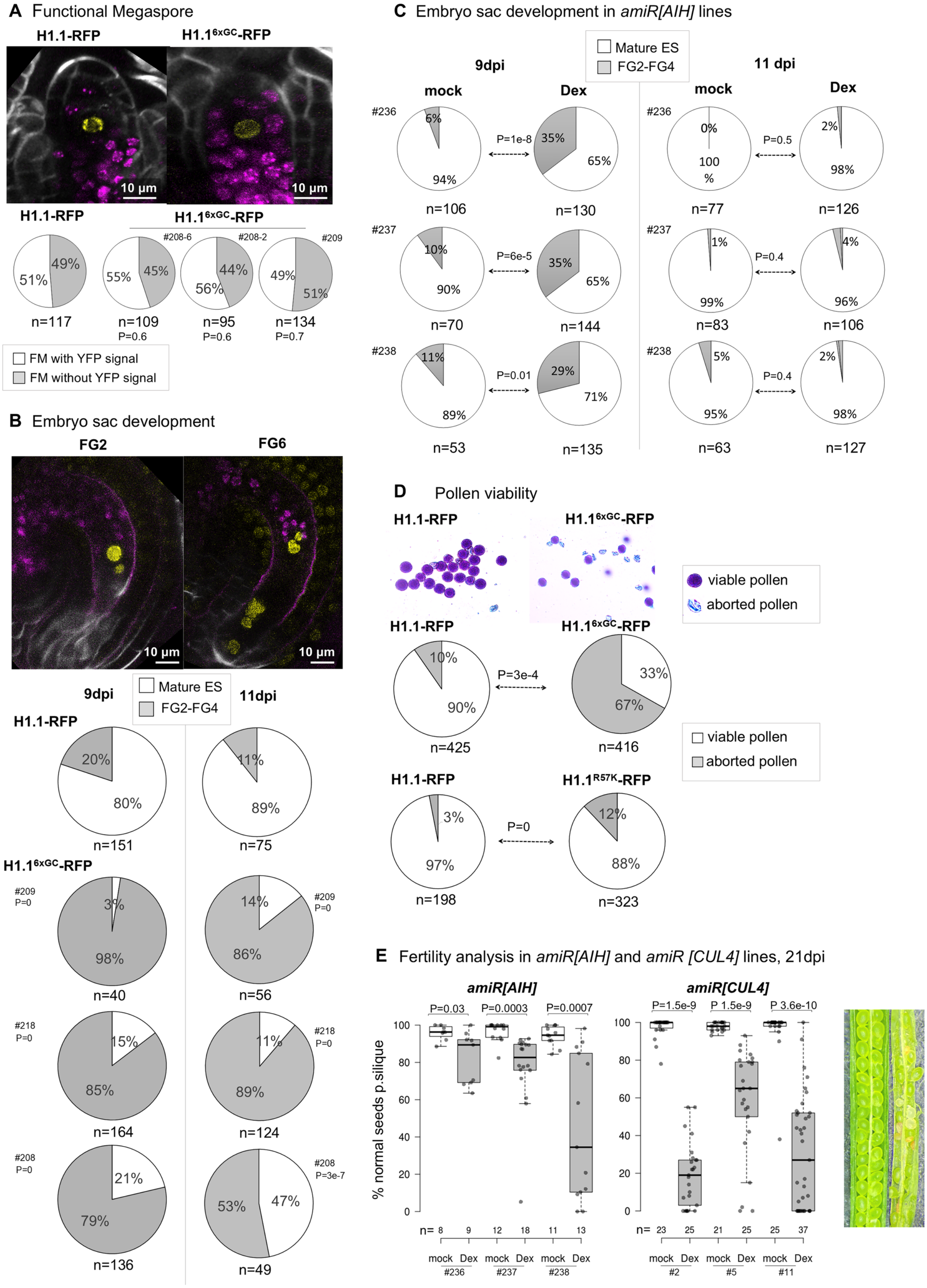
A Functional Megaspore
H1.1-RFP
H1.1^6xGC-RFP
10 µm
10 µm
H1.1-RFP
H1.1^6xGC-RFP
#208-6
#208-2
#209
49%
51%
55%
45%
44%
56%
49%
51%
n=117
n=109
P=0.6
n=95
P=0.6
n=134
P=0.7
FM with YFP signal
FM without YFP signal
B Embryo sac development
FG2
FG6
10 µm
10 µm
9dpi
Mature ES
FG2-FG4
11dpi
H1.1-RFP
20%
80%
n=151
11%
89%
n=75
H1.1^6xGC-RFP
#209
P=0
3%
98%
n=40
14%
86%
#209
P=0
n=56
#218
P=0
15%
85%
n=164
11%
89%
#218
P=0
n=124
#208
P=0
21%
79%
n=136
#208
P=3e-7
53%
47%
n=49
C Embryo sac development in amiR[AIH] lines
Mature ES
FG2-FG4
9dpi
11 dpi
mock
Dex
mock
Dex
#236
6%
94%
P=1e-8
35%
65%
n=106
n=130
#236
0%
100 %
P=0.5
2%
98%
n=77
n=126
#237
10%
90%
P=6e-5
35%
65%
n=70
n=144
#237
1%
99%
P=0.4
4%
96%
n=83
n=106
#238
11%
89%
P=0.01
29%
71%
n=53
n=135
#238
5%
95%
P=0.4
2%
98%
n=63
n=127
D Pollen viability
H1.1-RFP
H1.1^6xGC-RFP
viable pollen
aborted pollen
H1.1-RFP
H1.1^6xGC-RFP
10%
90%
P=3e-4
33%
67%
n=425
n=416
viable pollen
aborted pollen
H1.1-RFP
H1.1^R57K-RFP
3%
97%
P=0
12%
88%
n=198
n=323
E Fertility analysis in amiR[AIH] and amiR [CUL4] lines, 21dpi
amiR[AIH]
amiR[CUL4]
P=0.03
P=0.0003
P=0.0007
P=1.5e-9
P 1.5e-9
P 3.6e-10
% normal seeds p.silique
100
80
60
40
20
0
n= 8 9 12 18 11 13
n= 23 25 21 25 25 37
mock Dex mock Dex mock Dex
#236 #237 #238
mock Dex mock Dex mock Dex
#2 #5 #11

**Figure EV5. Impact of H1.1 persistence in the SMC on embryo sac development and fertility (related to Fig. 5).**

(**A**) Representative images and scoring of ovule primordia at 7 dpi expressing *AKV::H2B-YFP* in the functional megaspore in the control or H1.1$^{6GC}$-RFP line (3 independent lines). The image for H1.1-RFP is reused from Fig. 5A for facilitating comparisons. (**B**) Representative images and scoring of ovules at 9 dpi and 11 dpi (two days after emasculation at 9 dpi) expressing *AKV::H2B-YFP* in the embryo sac (stages FG2 and FG6 are shown) in the control line and scoring of these classes in the control and H1.1$^{6GC}$-RFP lines (3 independent lines). (**C**) Scoring of FG2-FG4 and FG6 embryo sacs identified by clearing, in ovules at 9 dpi and 11 dpi (two days after emasculation at 9 dpi) following the induction of *amiR[AIH]*. (**D**) Assessment of pollen viability by Alexander staining in the control and mutant lines as indicated, by scoring in anthers at 9 dpi. (**A–E**) *P* values, Fisher exact test. (**E**) Fertility analysis by quantifying the % of normal (green, plump) seeds per silique at 21 dpi after mock or dex treatment, in mutant lines as indicated, in three independent lines. *n*, number of siliques scored. *P* values from a Mann–Whitney U test. Boxplots: Center lines indicate medians; boxes span the interquartile range (25th–75th percentiles); whiskers extend 1.5× the interquartile range beyond the box limits, as computed in R. See also Source data Fig. EV5 and Table EV1. Source data are available online for this figure.

 © The Author(s)

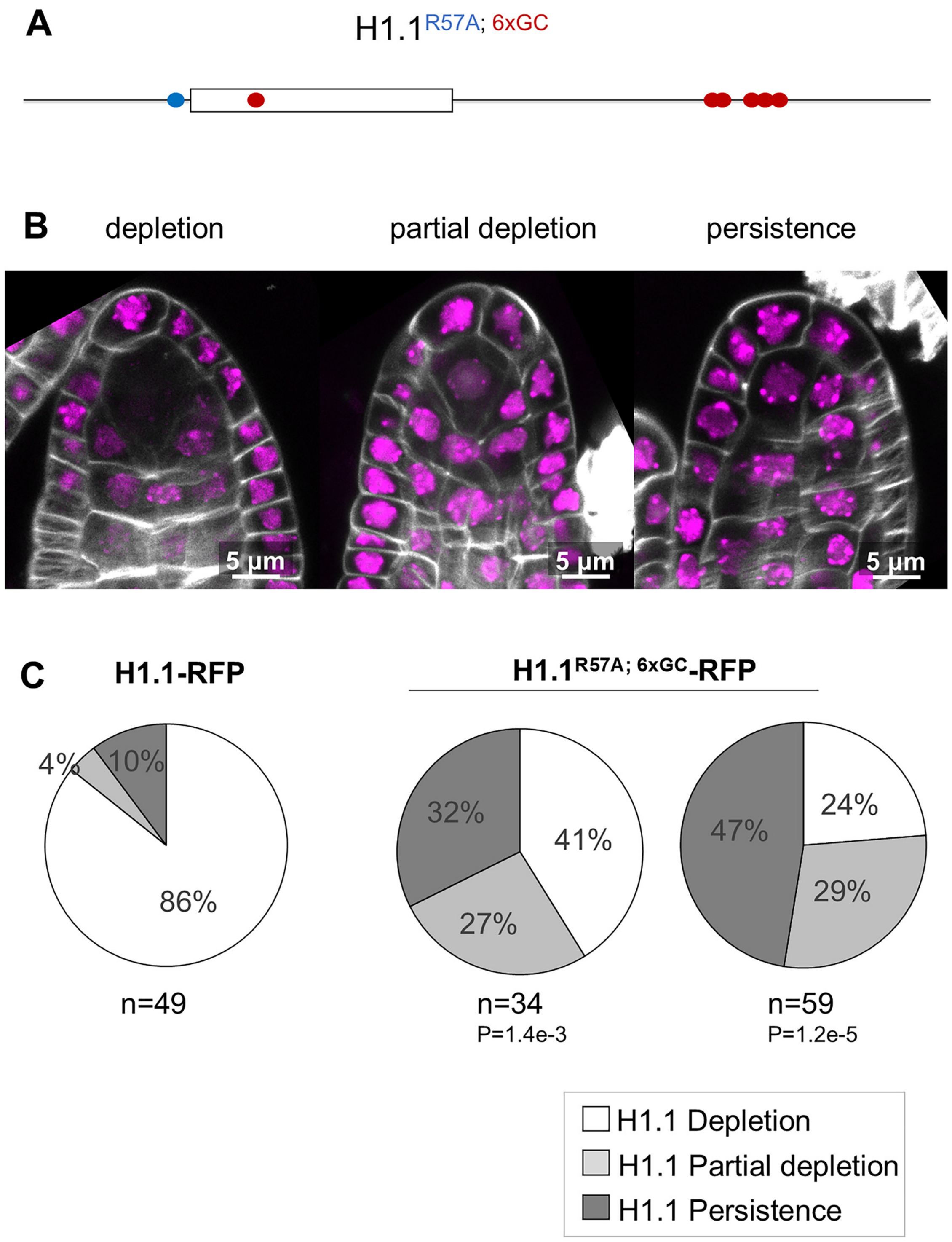


**Figure EV6. An H1.1 variant combining the R57A and K89R mutations shows resistance to degradation.**

(**A**) Schematic representation of the H1.1$^{R57A;6xGC}$ mutant variant showing the mutated R57A residue (blue) and the 6 K-to-R substitutions (red) among which K89R in the globular domain (box). (**B**) Representative images of the depletion, partial depletion and persistence phenotype as scored in (**C**). Note the diffused pattern in the 'partial' category indicating increased dissociation from heterochromatin suggested to be a result of R57A. (**C**) Scoring in one control line and two independent double mutant lines as indicated. *n*, number of ovule primordia scored at 5 dpi. *P* values, Chi-square contingency test of the three categories (depletion, partial depletion, persistence) distribution. See also Source data Fig. EV6 and Table EV1. Source data are available online for this figure.

© The Author(s)
